# Sleep Disorders in Childhood Neurogenetic Disorders

**DOI:** 10.3390/children4090082

**Published:** 2017-09-12

**Authors:** Laura Beth Mann Dosier, Bradley V. Vaughn, Zheng Fan

**Affiliations:** Department of Neurology, University of North Carolina at Chapel Hill, Chapel Hill, NC 27599, USA; vaughnb@neurology.unc.edu (B.V.V.); zheng_fan@med.unc.edu (Z.F.)

**Keywords:** Neurogenetic, Sleep, Neurodevelopmental, Angelman, Down syndrome, Trisomy 21, Smith–Magenis, Muchopolysaccharidosis, Achondroplasia, Duchenne, Congenital Central Hypoventilation

## Abstract

Genetic advances in the past three decades have transformed our understanding and treatment of many human diseases including neurogenetic disorders. Most neurogenetic disorders can be classified as “rare disease,” but collectively neurogenetic disorders are not rare and are commonly encountered in general pediatric practice. The authors decided to select eight relatively well-known neurogenetic disorders including Down syndrome, Angelman syndrome, Prader–Willi syndrome, Smith–Magenis syndrome, congenital central hypoventilation syndrome, achondroplasia, mucopolysaccharidoses, and Duchenne muscular dystrophy. Each disorder is presented in the following format: overview, clinical characteristics, developmental aspects, associated sleep disorders, management and research/future directions.

## 1. Introduction

Neurogenetic disorders represent a wide spectrum of diseases defined as “clinical diseases caused by a defect in one or more genes, which affects the differentiation and function of the neuroectoderm and its derivates” [1]. Our understanding and treatment of many human genetic diseases, including neurogenetic disorders, have been transformed by many genetic advances in the past few decades. In 2017, the total number of entries for genetic diseases in the Online Mendelian Inheritance in Man (OMIM) website was 24,061. It is estimated that as many as one-third of genetic disorders are primarily neurologic or have important neurologic involvement. Most neurogenetic disorders are classified as “rare disease.” The overall prevalence of neurogenetic disorders in the general population is not known; however, the estimated minimum prevalence in Northern England is 1 in 1100 of the population [2]. Collectively, neurogenetic disorders are not rare and are commonly encountered in general pediatric practice.

Sleep disturbances are extremely common in children with neurodevelopmental disorders. Approximately 25% of typically-developing, preschool-aged children have sleep related problems, whereas the prevalence of sleep disorders in children with neurodevelopmental disorders can be as high as 80% [3,4]. This high rate of sleep disorders is somewhat intuitive given that sleep is a multifaceted function of the central nervous system. The etiology of sleep disturbances in patients with neurogenetic disorders can include intrinsic neurologic pathophysiology, behavioral disorders, psychiatric disorders, medication side effects, and sleep disordered breathing. Regardless of etiology, sleep disturbances can have a significant impact on quality of life for both patients and caregivers [5,6,7,8]. Identification and treatment of sleep related disorders may significantly improve cognitive function or delay progression of underlying diseases for some patients with neurogenetic disorders [6,9,10,11,12]. It is important to understand the evaluation and management of sleep disorders related to pediatric neurogenetic disorders to improve patient and caregiver quality of life.

It is not practical to include sleep disorders in all neurogenetic diseases in this review article. The authors decided to select eight relatively well-known, neurogenetic disorders to provide a high-yield review of disorders that are commonly encountered in general pediatric practice or that have sentinel and specific features of sleep disorders that should be addressed during comprehensive care visits. Selected neurogenetic disorders were classified in Table 1 by the proposed location of the neurologic defect that impacts sleep. Each disorder is presented in the following format: overview of genetics including inheritance patterns, diagnosis and molecular basis, clinical characteristics with a focus on sleep, developmental aspects, associated sleep disorders (as they relate to sleep architecture, circadian rhythm disturbances, sleep related breathing, nocturnal movements or events, management of sleep-related disorders), and finally, research or future directions.

## 2. Review of Disorders

### 2.1. Down Syndrome

#### 2.1.1. Overview of Genetics

Down syndrome (DS) or trisomy 21 is the most commonly inherited genetic cause of intellectual disability and occurs in 1/650–1000 live births (OMIM #190685). In most cases of trisomy 21, the patient has three copies of chromosome 21. In 5% of individuals, one copy of chromosome 21 is translocated to another acrocentric chromosome, and, in 2–4% of cases, there is a mosaicism of trisomy (OMIM #190685). Mosaicism refers to the presence of more than one cell population present within the same individual. Depending on when non-disjunction of chromosomes occurred in germ-line or embryologic cell development, patients may have some cells with two copies of chromosome 21 and some cells with trisomy of chromosome 21 [13,14]. Disease presentation can be variable depending on the percentage of cells that express the genetic abnormality as compared with the percentage of normal cells.

#### 2.1.2. Diagnosis and Molecular Basis

Second trimester screening of serum alpha-feto protein is used to screen pregnant women for pregnancies complicated by trisomy 21. Recently, genetics from first-trimester fetal cells found in maternal plasma can be obtained for analysis, but caution should be taken in interpretation of results for trisomy 21 as these results have unclear specificity and sensitivity. Specific molecular basis of this multi-system disease is unknown. Chromosome 21 is a small chromosome that contains 225 genes [15]. A small segment on chromosome 21 (21q22.13) was proposed as the critical region responsible for the phenotype of DS [16]. One suggested region of interest includes the DS critical region (*DSCR*) on chromosome 21, which has been identified in the brain, heart, and tongue (OMIM #190685). It has been proposed that the expression of the *DSCR5* gene (OMIM #605938) within this critical region of 21q22.13 may contribute to the tongue malformation in DS, which has implications for sleep disordered breathing [17].

#### 2.1.3. Clinical Characteristics

Clinical characteristics of DS include characteristic facies with midface hypoplasia, narrow nasopharynx, narrow oropharynx, relative macroglossia, retroglossia, and adenotonsillar hypertrophy, which may predispose them to sleep disordered breathing. Hypotonia in younger children and increased body mass index in older children may also impact sleep related breathing. Additional characteristics include intellectual disabilities, hearing loss, increased risk of leukemias, cardiac malformations notably atrioventricular septal defects, Alzheimer’s disease, hypothyroidism, and gastrointestinal anomalies (OMIM #190685) [18].

##### Developmental Aspects

Intellectual disability in patients with DS can vary from mild to severe. Young patients with DS may have hypotonia that contributes to poor motor development, feeding difficulties during infancy, and sleep disordered breathing. Older patients with DS are at risk for early onset Alzheimer disease.

#### 2.1.4. Sleep Disorders

##### Sleep Complaints 

Sleep problems in children with DS include snoring, witnessed respiratory pauses, sleep disordered breathing, delayed sleep onset, frequent nighttime awakenings, excessive daytime sleepiness, and bed wetting. Sleep difficulties in children with DS may be more severe in patients with increased intellectual disabilities, and sleep related disorders could exacerbate cognitive and psychological dysfunction [9].

##### Sleep Architecture

Although overall sleep architecture is not vastly different from normal, children with DS have been shown to have decreased percent time spent in rapid eye movement (REM) sleep when compared with controls prior to airway intervention. Although percentage of REM sleep increased following airway intervention, it did not normalize when compared with controls [19]. They also demonstrate increased slow wave sleep and decreased sleep efficiency [20].

##### Sleep Related Breathing

Children with DS are at risk for both obstructive and central sleep apnea (CSA) [21]. These patients appear to have more central apneas as infants and young children while the obstructive features appear more frequently as the children age. Obstructive sleep apnea (OSA) may affect up to 50–79% of children with DS [14,18,19]. Signs and symptoms of sleep disordered breathing in patients with DS include heavy breathing, snoring, restless sleep, frequent arousals, daytime sleepiness, apneic pauses, and behavior problems [14]. Patients with comorbidities such as hypothyroidism or cardiac anomalies may be more likely to have severe OSA [18]. In a study of 34 non-obese children with DS, the mean obstructive apnea hypopnea index (oAHI) was within the severe OSA range and three- or four-month post-operative AHI following airway intervention (including adenoidectomy, tonsillectomy, and adenotonsillectomy) demonstrated reduction in OSA severity to the mild range. Although 17% of children with DS had resolution of OSA, 47% had persistent OSA, and 24% had increased severity of OSA [18]. In addition to OSA, hypotonia in the youngest patients may contribute to sleep related hypoventilation [21].

**Figure 1:**
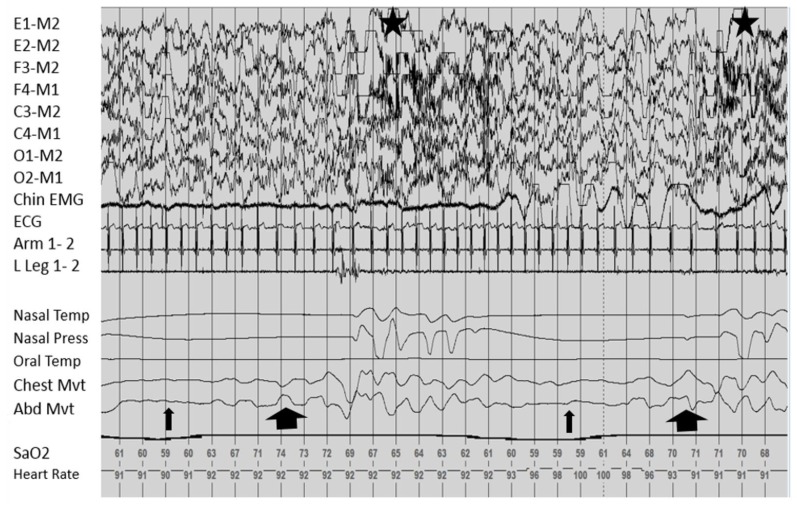
Frequent mixed apneas in a patient with trisomy 21. This figure, from a polysomnogram obtained from a child with trisomy 21, shows repetitive mixed apneas with the central portion of the apnea delineated by the thin arrow and obstructive component indicated by the thick arrow. Note the absence of airflow in the nasal and oral channels. Each event is associated with an arousal, noted by the stars.

In our recent study “Sleep Apnea and Hypoventilation in Patients with Down Syndrome: Analysis of 144 Polysomnogram Studies”, sleep disordered breathing, including hypoventilation, was common in patients with DS [21]. The obstructive component increased significantly with age and body-mass index (BMI), while the central component was prominent in the very young age group. Due to the overlap in progression of central and sleep apneas, mixed apneas are possible and represented in Figure 1. Due to the elevated risk of hypoventilation, which has not been previously highlighted, it may be helpful to consider therapies that target both apnea and hypoventilation in this population.

##### Movements and Nocturnal Events

Parasomnias have frequently been reported in children with trisomy 21 and likely improve with age [9]. Some parasomnias may be exacerbated by sleep related breathing disorders.

#### 2.1.5. Management

Children with trisomy 21 can require closer clinical monitoring for sleep issues. The majority of recommendations focus on the presence of sleep apnea. Current American Academy of Pediatrics (AAP) practice guidelines recommend discussing with parents the signs and symptoms of sleep apnea in the first six months of life and at each well-child visit. Patients with positive screening should be referred to a specialist with experience in managing pediatric sleep related breathing disorders: pulmonology, otolaryngology (ENT), sleep, or neurology [14]. While it is important to discuss sleep symptoms with parents, there is poor correlation between parental report of symptoms and polysomnogram results. Therefore, AAP guidelines recommend referral for a polysomnogram by four years of age in a center that routinely performs pediatric polysomnography [14]. Additionally, the comorbidity of obesity as a risk factor for sleep apnea should be discussed. Patients with OSA and DS should be evaluated by ENT for possible surgical interventions to maintain airway patency such as tonsillectomy, adenoidectomy, or adenotonsillectomy. Assessment of severity of OSA in patients with DS with a pre-operative polysomnography should be obtained. Overnight hospitalization following airway procedures is recommended particularly in patients with severe obstructive disease (an overall AHI ≥ 10 or significant oxygen desaturations associated with respiratory events) [22]. Post-operative sleep study should be obtained following any interventions to assess for improvement in severity and assessment for residual disease despite intervention. Patients with DS are at elevated risk for needing further intervention and treatment of OSA such as lateral positional therapy with a positioning device or positive pressure therapy with continuous or bi-level positive airway pressure (CPAP or BiPAP). Although studies in normal adults suggest treatment of OSA may slow progression of neurocognitive decline or development of cardiopulmonary disorders, no long-term studies have been performed in these patients. Given developmental challenges in trisomy 21, treatment plans should be tailored for the individual patient to account for developmental level, age, barriers to treatment, and comorbid conditions. For therapies that require patient compliance, the authors have found that these children respond to positive, non-food, reward-based behavioral approaches to improve patient participation.

#### 2.1.6. Research and Future Directions

Given the significant contribution of sleep disordered breathing to poor sleep quality in children with DS and the difficulties with tolerance of available therapies, more research should go into characterizing patients at the highest risk for development of sequelae of sleep disordered breathing. Development of novel interventions may improve tolerance and compliance with therapies to promote airway patency. 

### 2.2. Angelman Syndrome

#### 2.2.1. Overview of Genetics

Angelman syndrome (AS) (OMIM # 105830) is caused by the absence or non-functioning of the maternal ubiquitin-protein ligase E3A gene (*UBE3A*, OMIM #601623) on chromosome 15q11.2-q13 and occurs in 1 in 12,000–20,000 individuals, affecting males and females equally [23,24,25]. There are four main genetic mechanisms for this disorder. Approximately 70% of cases result from de novo maternal deletions of the gene, 7% result from paternal uniparental disomy of the gene, 1–2% from imprinting defects, 11% from mutations in the *UBE3A* gene, and the remaining 10% of individuals with classic phenotypic features of AS have negative genetic testing results [23,26]. Genetic abnormalities because of maternal deletions and paternal uniparental disomy have a low recurrence risk, but recurrence of disease in a sibling may be as high as 50% if the proband has a gene mutation.

#### 2.2.2. Diagnosis and Molecular Genetics

The mechanism of seizure activity in patients with AS has been attributed to UBE3A loss causing dysregulation of synaptic gamma-aminobutyric acid (GABA)ergic neurotransmission by deficient recruitment of functional GABA-A receptors [26]. This leads to thalamo-cortical dysfunction. The decrease in GABA-A receptors may induce an increase in thalamic GABA-B inhibition and cause a dysregulation of the thalamic pacing of GABAergic reticular neurons. These changes may contribute to the characteristic seizure activity and sleep architecture disruptions observed in AS [25]. The electroencephalogram (EEG) waveforms in AS are usually quite abnormal and consist of variable frequency (mostly in delta but can be in theta range), high amplitude, rhythmic activity mixed with spikes regardless of the presence of epilepsy. EEG findings, when seen in appropriate clinical context, can help to identify AS patients at an early age [27].

#### 2.2.3. Clinical Characteristics

Characteristics of AS include severe intellectual disability, recurrent seizures, ataxia, lack of speech, frequent laughter, jerky and puppet-like movements, disorders of sleep, and disorders of gait and balance. Epilepsy is one of the most common features and affects 80–90% of individuals. Typical craniofacial features include microcephaly, mid-facial hypoplasia, deep set eyes, a prominent pointed mandible, and skin and hair hypopigmentation [28].

##### Developmental Aspects

Patients exhibit significant intellectual disability, lack of speech, and most experience recurrent seizures consistent with epilepsy. The EEG pattern of rhythmical slowing mixed with spikes can be characteristic of AS, but detailed review of the variety of EEG patterns exceeds the scope of this review [25,28].

#### 2.2.4. Sleep Disorders

##### Sleep Complaints

The prevalence of sleep problems in AS ranges from 20% to 80%. Sleep problems appear to be most severe in early childhood between 2–6 years of age and may diminish or disappear by late childhood [24]. Individuals with AS have difficulty initiating and maintaining sleep, multiple nocturnal awakenings, difficulties falling asleep, reduced total sleep time, sleep–wake rhythm disorders, nocturnal movement disorders, enuresis, bruxism, sleep terrors, sleep walking, sleep paralysis, excessive daytime sleepiness, and sleep related breathing difficulties [28,29]. The presence of epilepsy was associated with endorsement of sleep problems. Frequent nighttime awakening was the most commonly reported sleep complaint in 80% of subjects. Parents reported 62% of children 2–14 years of age awaken more than two times per night, sometimes with prolonged awakenings. The frequency of such awakenings was shown to decrease with age [29]. Poor sleep quality and continuity can negatively impact neurocognitive development, social interactions, and behavioral disturbances which has been shown to impact caregiver stress and quality of life [5]. In fact, as compared with parents of children with other rare genetic conditions, parents of children with AS have reported higher levels of psychological distress including depression, stress, and anxiety [6].

##### Sleep Architecture 

The fragmented and reduced sleep in patients with AS is likely intrinsic to their severe encephalopathy. Children with AS experience difficulties settling at night and increased latency of sleep onset. It is unclear if this effect is due to abnormalities in circadian rhythm, melatonin production, or sensitivity. Significant investigation has characterized abnormal EEG characteristics of patients with AS that are present during wake and sleep. These underlying changes can make sleep staging on polysomnography difficult to interpret given the overlap between characteristic EEG features in AS and characteristic EEG sleep stage findings [30]. EEG characteristics include few changes in between wakefulness and stage 1 of sleep, making the transition from wake to sleep difficult to identify. Sleep spindles and K complexes, typically present in stage 2 sleep, were reduced, but these can also be difficult to identify due to slow rhythmic activity often present on EEG in children with AS. Additionally, notched, slow wave activity may be present in the central and posterior regions mixed with spike and wave activity in AS children less than four years of age during both awake and sleep periods. It may make stage N3 difficult to distinguish from awake state and epileptic activity [30]. The proportion of stage 2 sleep was not reduced as compared with children with non-specific intellectual disability, but the percentage of REM sleep was reduced. Reduced REM sleep persisted in children older than eight years of age with AS [24,25]. Additionally, there was increased percentage of slow-wave sleep [25]. There have been associations established between EEG findings and genotypes in AS. There have even been attempts to utilize EEG features to predict genotypes in AS [30]. Sleep-wake transition disorder was found in one study demonstrating hypnic startles and rhythmic movement disorders [28]. Nocturnal seizures may cause sleep fragmentation and REM sleep suppression thus making polysomnography difficult to interpret. Seizure activity can certainly disrupt sleep architecture and quality and worsen sleep deprivation which may, in turn, enhance epileptic activity. Many children with AS are prescribed anti-epileptic medications, which may decrease the epileptic disruption or may independently cause changes to sleep architecture. In summary, sleep stages may be difficult to differentiate due to the frequent presence of abnormal EEG background in patients with AS.

##### Sleep Related Breathing 

There is little polysomnographic data on sleep disordered breathing in patients with AS due to poor tolerance of the laboratory study in these children with severe intellectual disability. In a questionnaire study of patients with AS, snoring was reported in 32% and sleep difficulties in 19% of patients [29]. Individuals with AS are at risk for central and obstructive sleep apnea, and a polysomnographic evaluation of ten children with AS revealed moderate to severe sleep apnea (both central and obstructive) in 30% of subjects. Presence of epilepsy was associated with an increased risk for moderate to severe sleep apnea [28].

##### Movements and Nocturnal Events

AS subjects demonstrated a trend toward a higher periodic limb movement index (PLMI) although the result was not significant [28]. The parents reported increased presence of nocturnal hyperkinesia, unusual movements during sleep, pain of unknown origin during sleep, nocturnal seizures, enuresis, bruxism (22%), night terrors (5–6%), and sleep walking as compared with typically developing controls. Sleep talking was reported less frequently than in controls [29]. Sleep related rhythmic movement disorders such as body rocking, body rolling, or head banging are present in both children with AS and normal subjects, but in children with AS these behaviors persisted beyond the expected age of 3–4 years [24].

#### 2.2.5. Management

Management of sleep related problems in AS should focus on the stabilization of the sleep-wake pattern and development of a consistent, well-ordered schedule. This can be difficult to attain, and frequently the difficult nights can occur in clusters. The goal of management is to identify triggers that precipitate clusters of difficulties and avoid them. One strategy is to develop a pre-bedtime regimen that can help accentuate the clues of upcoming bedtime. Additionally, limiting stimulating factors in the bedroom may be beneficial. Behavioral management of sleep related problems is the mainstay of treatment. Basic principles of behavioral modification involve educating and empowering the parent to create a sleep-compatible environment, adjust and regulate the sleep–wake schedule to consolidate sleep, promote independent sleep initiation, and manage parent–child interactions to reinforce appropriate child behavior [5]. Patients with AS were found to have lower nocturnal serum melatonin level than controls [31]. In one uncontrolled study of the use of low-dose melatonin (0.3 mg) in children with AS, wrist actigraphy recorded decreased motor activity and increased total sleep time for six days. The maintenance of this effect was not studied [32]. A larger study of melatonin in children with intellectual disability (one patient with AS) reported decrease in sleep onset latency, but melatonin did not influence the number of nighttime awakenings or total sleep duration. The patient with AS required relatively high doses of melatonin (9 mg) [24]. Lastly a randomized, double-blinded placebo control trial of melatonin in AS found that melatonin decreased sleep latency, increased total sleep time, and decreased nighttime awakenings, but salivary melatonin became very high after a few weeks [33]. For the management of parasomnias, the bedroom environment should focus on prevention of injury associated with sleep walking by limiting the height of the bed, obstructing access to windows, and removing features that may cause injury [24]. These patients also may have seizures at night and require higher anticonvulsants doses at night.

#### 2.2.6. Research and Future Directions

As discussed, melatonin has been suggested as an effective therapy for promoting sleep onset and maintenance in AS, but the evidence is weak due to small patient samples; however, if melatonin is efficacious, it implies that circadian rhythm dysfunction may play a role in the sleep problems of AS. Further investigation into optimal dosing and which patients would be best treated with melatonin would be helpful [24]. Finally, understanding more clearly the contribution of GABAergic dysregulation on sleep homeostasis in AS may help elucidate novel therapeutic targets for both sleep and epilepsy treatment [29].

### 2.3. Prader–Willi Syndrome

#### 2.3.1. Overview of Genetics

Prader–Willi syndrome (PWS) (OMIM #176270) is caused by absence or non-functioning of the paternally derived genes at 15q11.2-q13 [34]. Notably, 65–75% of cases are caused by deletion of this region on the paternally-inherited chromosome. Furthermore, 20–30% of cases are caused by maternal uniparental disomy, 1–3% of cases are caused by a methylation and imprinting defect, and less than 1% of cases due to paternal translocation [34,35]. More recent data suggests that contribution of paternal deletion may be decreasing and maternal disomy may be increasing due to increased maternal age at conception [36]. PWS occurs in 1/10,000 to 1/25,000 live births [37]. Most cases result from de novo, sporadic mutations, and risk of recurrence is the same as the general population; however, in cases of an imprinting defect, the father of the child should be evaluated as a germline carrier of the deletion which could result in a 50% recurrence risk, if present. The risk of recurrence of chromosome translocations can be up to 10% [34].

#### 2.3.2. Diagnosis and Molecular Genetics

Potential genes involved in the region of DNA affected by PWS include the small nuclear ribonucleoprotein polypeptide N gene (*SNRPN*, OMIM #182279) and the necidin gene (*NDN*, OMIM #02117). The *SNRPN* gene encodes a splicing factor involved in RNA processing and an upstream reading frame polypeptide. The *SNRPN* gene shows highest expression in the brain and heart. In mice, necidin is a protein encoded by neural differentiation specific mRNA, and expression is found in human brain, fibroblasts, and placenta.

In suspected cases of PWS, DNA methylation testing from peripheral lymphocytes should be the initial diagnostic test. If further testing is required, fluorescence in situ hybridization (FISH), high-resolution chromosome analysis (DNA microarray), DNA methylation analysis can detect genetic abnormalities; however, FISH alone will not distinguish PWS from AS. Although genetic testing is now widely available, not all obese children with learning disabilities need to be tested for PWS. If findings of core features are not seen such as hypotonia, failure to thrive, and undescended testes in boys, then genetic testing for PWS will likely be negative [34].

#### 2.3.3. Clinical Characteristics

The clinical features of PWS are age-related. *In utero*, fetuses demonstrate decreased movement and/or polyhydramnios. In infancy, patients demonstrate hypotonia, hypogonadism (cryptorchidism), feeding difficulties, failure to thrive, and developmental delay [37,38,39]. As children age, hypothalamic-pituitary dysfunction is thought to contribute to hyperphagia, obesity, inefficient growth hormone (GH) secretion, adenotonsillar hypertrophy, and sleep disordered breathing. Unfortunately, young patients with PWS have an increased risk of sudden death, particularly during sleep. A large portion of these occurrences have been attributed to respiratory disorders including respiratory infections and sleep disordered breathing. Central adrenal insufficiency and hypothalamic-pituitary dysfunction may also contribute to these events [39]. Other physical attributes of some patients with PWS include hypopigmentation (particularly in patients with deletions), thin upper lip, almond-shaped eyes, bitemporal narrowing, short hands with tapered fingers, and small feet [34].

##### Developmental Aspects

In addition to hypotonia and motor delays, infants and young children with PWS may demonstrate feeding difficulties early in infancy, cognitive impairments in the mild to moderate range, features of autism, and aggression. Psychological problems such as temper tantrums and obsessive behaviors becoming more evident in later childhood and adolescence [34,38]. Some of the neurodevelopmental components of PWS could be exacerbated by the presence of obstructive sleep apnea [10,11,12].

#### 2.3.4. Sleep Disorders

##### Sleep Complaints

Primary sleep complaints in PWS include sleep disordered breathing, snoring, excessive daytime sleepiness (EDS), and hypoarousability during sleep [36,38,39,40]. In addition, 60–80% of patients with PWS are reported to have EDS, and severity may increase with age. In PWS, obstructive sleep apnea is unlikely to fully explain EDS and hypothalamic dysfunction likely contributes to symptoms [36,41]. Parents report reduced sleep onset latency at night and during the day. Excessive sleepiness in PWS seems to be increased when compared to other groups with intellectual disabilities [36]. There is debate as to whether EDS is associated with specific genotype [36]. One study with a total of eight patients reports increased EDS in the patients with paternal deletion as compared to maternal uniparental disomy [42], whereas another group that studied 17 children with PWS reported no association between EDS and genotype [43]. There is also conflicting evidence on whether sleep disordered breathing or apneas have a genotype association [36]. Narcoleptic type symptoms such as sleep onset rapid eye movement (SOREM) sleep, cataplexy, and sleep attacks have also been shown to have increased frequency in patients with PWS compared with other neurodevelopmental disorders [36,43,44].

##### Sleep Architecture

On review of 13 studies of Multiple Sleep Latency Tests (MSLT) in PWS, eight studies confirmed reports of EDS with shortened sleep latency of less than 5 min on objective measure by MSLT [36]. Additionally, several studies have suggested that disorders in sleep architecture may contribute to excessive daytime sleepiness due to the abnormal presence of REM sleep at sleep onset (SOREM in the first sleep stage period), reduced latency to REM onset (<70 min), and fragmented REM sleep [36,41,43,45,46]; however, a separate study by Verillo et al. identified an increase in the percentage of stage 1 sleep, a decrease in slow wave, and a decrease in REM sleep [47]. In a review of studies reporting REM abnormalities, PWS patients with and without obstructive sleep apnea were found to have REM abnormalities [36]. Human leukocyte antigens (HLA) haplotypes have been associated with narcolepsy in the general population. Genetic abnormalities associated with altered HLA expression have not been found at an increased rate in patients with PWS [36,41,44]. Although insufficient evidence has been found to associate circadian melatonin excretion abnormalities with sleep architecture abnormalities in PWS, there is growing evidence for contribution of hypothalamic dysregulation by hypocretin/orexin and ghrelin [36].

##### Sleep Related Breathing

Factors impacting sleep disordered breathing (SDB) in infants and young children with PWS include facial dysmorphism, small nasopharynx, and small oropharynx with or without adenotonsillar hypertrophy. Hypotonia and/or scoliosis lead to restrictive lung disease and sleep-related hypoventilation [38]. Increased CSA has also been reported in PWS. Hypothalamic dysfunction and blunted chemoreceptor sensitivity likely contribute to altered central respiratory control in response to hypoxemia and hypercapnia particularly in very young children [38,48]. In pre-clinical studies of necidin gene deletion, mice demonstrated abnormal ventilatory control with prolonged central apneas [48]. In one study of 28 infants with PWS, a majority of the infants were diagnosed with CSA; however, on follow up sleep study at two years of age, most, but not all, patients exhibited resolution of CSA [37]. In this same study of infants, the obstructive apneahypopnea index was low and most of the obstructive events were documented as hypopneas, which may be due to baseline hypotonia. One suggested mechanism for this phenomenon is that due to hypotonia and decreased respiratory muscle strength, infants with PWS have a lower resting functional residual capacity (FRC) than normal infants, putting them at risk for sleep related hypoventilation particularly during REM sleep [48].

Due to the increased risk of obesity with age, patients with PWS should continue to be assessed for OSA. OSA is frequently reported in patients with PWS. Though much of the evidence is mixed regarding this association, most of the studies have included both adult and pediatric subjects [36]. Evaluation of airway obstruction with endoscopy in nine children with PWS and OSA revealed multi-level upper airway obstruction [35]. One study of 30 children (mean age seven years old) with PWS found an association between obesity and severity of obstructive apnea-hypopnea index [40], but several smaller studies did not identify this association [35]. Due to the increased risk for sudden death in infants and young children less than three years of age with PWS polysomnography is recommended in infancy, both prior to growth hormone treatment and monitoring during growth hormone therapy [37,38,49].

#### 2.3.5. Management

In infants with PWS and CSA, supplemental oxygen therapy was shown to decrease central apnea index on polysomnography and prevent desaturation events [48]. If supplemental oxygen is insufficient in reducing sleep related hypoxemia associated with central apneas, some patients may require BiPAP [37]. As previously, discussed central apnea typically, but not always, improves with age. Although CSA may improve, obstructive sleep apnea and sleep-related hypoventilation may develop.

Growth hormone treatment in PWS has been shown to improve hypotonia, muscle strength including respiratory muscles, linear growth velocity, and cognitive performance, while decreasing total body fat [34,37]. There has been some evidence suggesting worsened upper airway obstruction due to increased adenotonsillar hypertrophy of patients receiving growth hormone therapy. There have been concerns about a possible increase in sudden death in young children and infants with PWS on growth hormone therapy, but, due to the overall benefits of growth hormone in PWS, this therapy is often recommended with increased monitoring by referral to otorhinolaryngology (ENT) and frequent monitoring of sleep-related breathing symptoms [34,38,49].

Adenotonsillectomy can be helpful in the reduction of upper airway obstruction and obstructive apnea-hypopnea index in patients with PWS, but, in many patients, particularly those with a severe OSA, it may not be sufficient. Children with PWS may be at increased risk for post-operative complications following adenotonsillectomy and should be monitored closely following surgery. Additionally, post-operative sleep study should be obtained [35,38,43,50]. Findings on airway evaluation that were associated with incomplete resolution of OSA following adenotonsillectomy include lingual tonsillar hypertrophy, tongue base obstruction, and airway hypotonia leading to laryngomalacia and/or pharyngomalacia [35]. Similarly, obesity may contribute to incomplete surgical correction of the OSA. Approximately 17–40% of PWS patients require positive airway pressure (PAP) initiation to treat residual OSA, and this rate appears to increase with age [38,43].

Modafanil is a medication that acts as a central stimulant of alpha-1 adrenergic receptors and is used to promote wakefulness. One open label study has evaluated the use of modafinil in nine patients with PWS and EDS without confounding OSA. Modafinil was well tolerated and improved sleepiness in all patients according to the Epworth Sleepiness Scale, but did not significantly reduce BMI [51].

#### 2.3.6. Research and Future Directions

As PWS is a very rare disease, most of the studies on the mechanisms underlying EDS, CSA, and OSA are unclear. In addition, it is unclear which patients are at highest risk of sudden death or treatment failures. More research is needed to determine genotype-phenotype associations, the effect of OSA on EDS, and effective treatments such as modafinil for daytime symptoms, weight loss strategies, and optimal treatment of sleep disordered breathing.

### 2.4. Smith–Magenis Syndrome

#### 2.4.1. Overview of Genetics

Smith–Magenis Syndrome (SMS) (OMIM #182290) is an autosomal dominant disorder usually caused by an interstitial deletion of 3.5 Mb on chromosome 17p11.2, which includes the Retinoic Acid Induced 1 (*RAI1* gene) typically due to chromosome recombination errors during meiosis [52,53,54,55]. These meiotic recombinations account for 70% of SMS, but 10% of patients with the SMS phenotype have heterozygous point mutation of *RAI1* (OMIM #607642). The birth incidence of SMS is 1/25,000, and all cases have appeared de novo [55].

#### 2.4.2. Diagnosis and Molecular Genetics

The *RAI* expression is primarily found in brain tissue. Mutations in *RAI1* have been associated with an inverted rhythm of melatonin secretion. RAI1 has been suggested to be a direct, transcriptional regulator of the circadian locomotor output cycles kaput gene (*CLOCK*) and that mutations in the *RAI1* gene cause altered expression of multiple downstream circadian genes including *PER2, PER3, CRY1*, and *BMAL1* [54]. Diagnosis is established with FISH or comparative genomic hybridization (CGH array). If FISH and CGH are negative, then sequencing of the *RAI1* gene should be obtained [53]. In one study, SMS was diagnosed on genetic deletion in 78% of individuals, genetic mutation in 18% of individuals, and clinical features alone in 4% of patients [52].

#### 2.4.3. Clinical Characteristics

Features of SMS include intellectual disability, behavioral problems, dysmorphic features, heart defects, and kidney defects. Dysmorphic features include light-colored hair, bulging forehead, midface or nasal bridge hypoplasia, cleft palate, micrognathia, ocular anomalies (hypertelorism, oblique outer and upper palpebral fissures, synophrys, iris anomalies, microcornea), tooth agenesis, high arched palate, hypoacousia (hearing loss), short stature, hoarse voice, vocal cord nodules, and scoliosis. Other congenital abnormalities include: congenital heart disease in 30% of patients (including cyanotic heart lesions), spleen and kidney malformations, hypothyroidism, hypercholesterolemia, and specific immunoglobulin deficiency. Lastly, many subjects are obese, and there has been evidence to suggest hyperphagia similar to patients with PWS [56].

##### Developmental Aspects 

Neurodevelopment is significantly affected in patients with SMS. During the neonatal period parents report infants being very calm and sleeping a lot but having difficulties with hypotonia and feeding. Parents may report unsteady gate and possibly hyporeflexia. Neurologic evaluation may reveal magnetic resonance imaging (MRI) findings of ventricular or cisterna magna enlargement or partial cerebellar agenesis, epileptic seizures, or asymptomatic seizure activity on EEGs. Stereotypical movements noted during development are self-hugging and the tendency to put one’s hands in one’s mouth. Although some patients have exhibited near normal intelligence quotient (IQ), almost all have intellectual or behavioral disabilities including speech delay, expressive language deficits, self-injury, aggression, outbursts, attention deficits, and hyperactivity. Behavioral difficulties can be exacerbated by speech difficulties and sleep disorders [53].

#### 2.4.4. Sleep Disorders

##### Sleep Complaints 

Parents report alterations in the sleep–wake cycle in patients with SMS. Parents report hypersomnolence during infancy, but later symptoms evolve into difficulty falling asleep, staying asleep, frequent nighttime awakenings several times per night, temper tantrums, multiple naps during the day, and sleep attacks toward the end of the day in early toddlerhood [53,54,55].

##### Sleep Architecture

Patients are reported to have early sleep onset, prolonged nocturnal periods of wakefulness, hyperactivity, early sleep offset, EDS, and decreased total sleep time [52].

##### Circadian Rhythm

Circadian rhythm disruption is a prominent feature of the sleep disorder in SMS. Etiology of these sleep effects are likely related to circadian rhythm disturbances of melatonin secretion. Typically, melatonin secretion is released by the pineal gland at the onset of darkness. Although there are gender- and age-related variations in patterns of melatonin secretion, the majority of subjects with SMS has increased daytime levels of salivary melatonin. It is unclear if there is true inversion of melatonin secretion or a shift in phase or timing of the cycle [52]. Typically, the circadian cycle is regulated by light-triggered retinal signals via the retinohypothalamic tract, and expression of circadian genes oscillates on a 24-h cycle. The precise involvement of the *RAI* gene expression is unclear [53].

#### 2.4.5. Management

Management of children with SMS should focus on reestablishing the circadian rhythm. Exogenous administration of melatonin has been employed as a treatment strategy to attempt to restore the nocturnal rhythm of sleep onset. Although patients have reported initial effectiveness of this strategy, there may be a subsequent loss of response. One suspected hypothesis for this is accumulation of melatonin with traditional dosing. Therefore, low dose regimens are suggested [53].

Melatonin release is controlled by the sympathetic nervous system and blocked by beta-adrenergic antagonists. A second treatment strategy for regulation of circadian rhythm in SMS uses beta-adrenergic antagonists administered in the morning to block daytime endogenous melatonin release in combination with timed evening exogenous melatonin administration to promote sleep onset. This treatment strategy has been shown to restore normal sleep habits, resulting in improved daytime behavior and quality of life for both patients and families [55,57]. Daylight is another clue used to help establish the circadian rhythm, but this has not been studied in this group.

#### 2.4.6. Research and Future Directions

The direct link between the gene mutations identified in SMS and the symptoms manifested by circadian rhythm disturbances has not yet been elucidated. Further research is needed to better understand this mechanism and provide insights for new therapeutic targets such as light therapy and management strategies.

### 2.5. Central Congenital Hypoventilation Syndrome

#### 2.5.1. Overview of Genetics

Congenital central hypoventilation syndrome (CCHS), previously known as Ondine’s curse, is an autosomal dominant disorder that results from a heterozygous mutation in the paired-like homeobox 2B gene (*PHOX2B*) on chromosome 4p13 in the majority of patients (OMIM #209880, #603851). The majority of CCHS cases (~95%) are due to polyalanine repeat expansion mutations (PARMs) and the minority (~5%) are due to non-polyalanine repeat expansion mutations (NPARMs).

#### 2.5.2. Diagnosis and Molecular Genetics

*PHOX2B* has two polyalanine repeat regions of trinucleotide repeats of one of four codons that code for the amino acid repeat or polyalanine repeat expansion mutations. The normal *PHOX2B* region in unaffected individuals has 20 alanines on both *PHOX2B* alleles. Generally, increased number of alanine repeats correlates with disease severity, but clinical expression of genotype can be variable. Individuals heterozygous for 24 alanine repeats and some with 25 alanine repeats may have very mild disease, but other individuals with 25 alanine repeats often present in the neonatal period with severe disease. The longest known repeat is 33 alanines. Patients with 26–33 alanine repeats have more severe disease [58]. Other *PHOX2B* variants may not have polyalanine expansions and may have alterations outside of the polyalanine repeat called non-polyalanine repeat expansion mutations (NPARMs), which is typically associated with a more severe phenotype. Of many reports of transmission of polyalanine repeats from a mosaic parent to their child, there was no change in the observed number of polyalanine repeats from parent to child [58]. PHOX2B is a tissue-specific transcription factor that regulates expression of a series of genes involved in the autonomic nervous system. PHOX2B binds directly to the regulatory regions of other genes such as dopamine-b-hydroxylase (*DbH*), *PHOX2A*, and T-Cell Leukemia Homeobox (*TLX-2*) regulating their expression, but the entire mechanism is not well described [59].

#### 2.5.3. Clinical Characteristics

Classic presentation of CCHS occurs in the newborn period with hypoventilation, disordered respiratory control particularly during sleep, and autonomic nervous system dysregulation. Features include breath holding spells, lack of normal ventilatory responses to exercise or environmental responses, diminished pupillary response, esophageal dysmotility, constipation, profuse sweating, reduced basal body temperature, and an altered perception of anxiety. Work up for primary neuromuscular, cardiac, pulmonary, or brainstem lesions do not identify a reasonable etiology. Conditions that can be associated with CCHS include Hirschsprung disease from abnormal migration of neural crest cells or tumors of neural crest origin such as neuroblastoma, ganglioneuroblastoma, and ganglioneuroma. Typical facial features include characteristic box-shaped face in which the face is short relative to the width, flattened face, and inferior inflection of the lateral third of the upper vermillion border (lip trait) [60]. Classically patients exhibit periods of apnea or hypoventilation during sleep, but, in more severe disease, episodes may occur while awake or asleep. The frequent sleep state of the newborn can make this difficult to determine. In addition, some individuals with mild disease may present with later onset of symptoms after one month of age including later childhood or adulthood [59].

##### Developmental Aspects

Most patients with CCHS do not have recognized neurocognitive dysfunction, but there have been reports of neurocognitive impairment in two patients with severe CCHS. One study of the Bayley indices of 31 pre-school children with CCHS demonstrated a mean Bayley index well below the normal mean for age, but it should be noted there was wide variability in scores of children with CCHS in this study [61]. A second study testing IQ in 12 children with CCHS noted that 2 of 12 patients with CCHS has an IQ less than 70, meeting the definition of intellectual disability [62]. Chronic hypoxemia and hypoventilation may impact the development of cognitive dysfunction. Therefore, prevention of decline through optimal management and routine neurocognitive screening should be performed [59]. 

#### 2.5.4. Sleep Disorders

##### Sleep Complaints

Newborns with CCHS are frequently identified by central apneas, hypoventilation, and hypoxemia in the newborn nursery. Most patients with CCHS have awake ventilation sufficient to maintain gas exchange; however, severely affected patients may experience hypoventilation during periods of wakefulness and sleep [59]. Periods of sleep and wakefulness can be difficult to distinguish and treat during the newborn period due to the sleep patterns of newborn infants. In a recent study, it has been shown that parents of children with CCHS (average age 9 years ± 6 years) experienced decreased sleep quality, increased daytime sleepiness, and increased depression indices when compared to parents of normal children despite reporting that there was not significant need for parental intervention overnight for children with CCHS. Minimal need for overnight caregiver intervention was surprising given that all CCHS patients in this study required positive pressure ventilation overnight (11 via facemask and 12 via tracheostomy) [63].

##### Sleep Architecture

Sleep disordered breathing is worsened during REM sleep in most disorders such as OSA because of decreased ventilatory drive and hypotonia of accessory respiratory muscles. In patients with CCHS, hypoventilation has been found to be more severe during non-REM sleep (especially stages N1 and N2) than REM sleep, which is similar to patients with central apnea with Cheyne–Stokes breathing. Breathing abnormalities in REM sleep may still be severe [64,65,66]. This is surprising since hypoventilation is uncommon while awake and passive limb movements during sleep have been shown to reduce hypoventilation in patients with CCHS, and normal subjects without CCHS are more easily aroused and have increased limb movements during non-REM sleep [66,67].

##### Sleep Related Breathing

Sleep disordered breathing in CCHS may range from alveolar hypoventilation during non-REM sleep and normal ventilation during wakefulness to severe complete central apneas during sleep and severe hypoventilation while awake. It has previously been shown that when patients with CCHS are administered hypercarbic gas, they have decreased ventilatory response than expected to CO_2_ [64,68]. On polysomnography, significant central apneas and severe hypoventilation are observed with severe hypercarbia with pCO_2_ above 45 mmHg and severe prolonged hypoxemia with oxygen saturation below 85%. The mainstay of treatment of these gas exchange abnormalities in children is mechanical ventilatory support to prevent the development of pulmonary hypertension and *cor pulmonale* [59,64,66,68].

#### 2.5.5. Management

The American Thoracic Society (ATS) published a clinical policy statement in 2010 about the diagnosis and management of CCHS [59]. Prior to diagnosis with CCHS, patients should be evaluated for cardiac, brainstem, and metabolic abnormalities. If genetic analysis is consistent with CCHS, then additional screening for pulmonary hypertension and cor pulmonale as sequelae of chronic hypoventilation should be obtained. Patients with CCHS should also be evaluated for cardiac arrhythmias and gastrointestinal manifestations including Hirschsprung disease. ATS guidelines recommend yearly screening with 72-h Holter cardiac monitoring, echocardiogram, hematocrit, reticulocyte count, and ophthalmologic exam. Children at increased risk of needing 24-h ventilation are children with NPARMs or PARMS 27–33 repeats in length. These severely affected patients should be screened annually for neural crest tumors with imaging.

ATS guidelines also recommend annual or biannual physiologic studies during wake and sleep and during spontaneous breathing or baseline home ventilatory support to titrate ventilatory support during growth and development of the patient. Modalities for ventilatory support in patients with CCHS include tracheostomy and ventilation, non-invasive BiPAP, negative pressure ventilation, and diaphragmatic pacing. Treatment with supplemental oxygen has been shown to be ineffective in treating hypoventilation, and patients are still at risk for pulmonary hypertension and cor pulmonale. As patients with CCHS can suffer severe respiratory arrest at sleep onset, they should be closely observed and assistive ventilation should be initiated prior to sleep onset. Hypoventilation has not been shown to improve with age and patients with CCHS are not candidates for weaning from ventilatory support.

Some children may be candidates for phrenic nerve or diaphragm pacing in which electrodes are implanted near the phrenic nerve or diaphragm and connected to an internally implanted receiver. An external pacer transmits pacing signals to the internal receiver. These devices are recommended for optimal ventilation. Benefits to this type of respiratory pacing are that ventilatory support can be increased for more hours of the day compared to mechanical ventilation used for sleep; however, for patients that require 24-h ventilation, tracheostomy for back up ventilation in the event of pacer failure is still recommended. There is increased risk of obstructive apnea in patients with diaphragmatic pacing due to asynchronous diaphragm contraction with upper airway skeletal muscle contraction. Improving technological support and aggressive management of patients with CCHS has led to a low mortality rate for CCHS. Most patients are living into adulthood with a good quality of life [59].

#### 2.5.6. Research and Future Directions

Given the heterogeneity of clinical presentations including age of onset and presence of various genotypes, more research is needed to identify genotype/phenotype correlations and further elucidate the mechanisms of CCHS. An improved understanding of the mechanism of disease is the first step in identifying novel therapeutics that may be able to take the place of mechanical ventilatory support. Diaphragm pacing is a relatively new therapy, further studies including long term outcomes and complications are needed particularly in children. Better understanding of autonomic dysfunction would help to guide screening and therapies to prevent sequelae of these physiologic disruptions.

### 2.6. Achondroplasia (Including Hypochondroplasia)

#### 2.6.1. Overview of Genetics

Achondroplasia is the most common inherited skeletal dysplasia with a birth incidence of one in 10,000–30,000 (OMIM #100800). Inheritance occurs in an autosomal dominant pattern due to a heterozygous, gain of function mutation in the fibroblast growth factor receptor 3 gene (*FGFR3*) on chromosome 4p16.3 (OMIM #134934). The point mutation substitution of an arginine for glycine at position 380 was identified in the *FGFR3* gene and is often a de novo mutation in 50% of the patients [69,70,71]. Hypochondroplasia is included here for discussion because it can also involve a mutation in the *FGFR3* gene with a similar but milder clinical phenotype and autosomal dominant inheritance. Not all patients with hypochondroplasia have identified mutations (OMIM #146000).

#### 2.6.2. Clinical Characteristics

Typical physical characteristics of individuals with achondroplasia are considered to be an effect of increased FGFR3 signaling on endochondral bone and include short stature, rhizomelic shortening of the ribs, frontal bossing, macrocephaly, midface hypoplasia, lumbar lordosis, limitation of elbow extension, genu varum, and trident hand [69]. Natural history studies have indicated higher mortality in children with achondroplasia as compared to the general population which has been attributed to foramen magnum stenosis (FMS), sudden death, and sleep disordered breathing [69].

##### Developmental Aspects

Although children with achondroplasia have been reported to have IQ within the normal range, in comparison with siblings, children with achondroplasia may have lower verbal comprehension and increased neuropshychological and cognitive deficits [71]. It is important to evaluate these children for sleep related abnormalities, as these neurodevelopmental functions can worsen related to poor quality sleep.

#### 2.6.3. Sleep Disorders

##### Sleep Complaints

Sleep disorders present early in life in patients with achondroplasia and are often underreported by parents particularly in patients three years of age or younger [72]. In one study which did evaluate parental complaints via questionnaire, parents reported increased incidence of snoring, apnea, mouth breathing, and open bite in preschool and school-aged children with achondroplasia compared with the control group [73].

##### Sleep-Related Breathing

There is an increased incidence of sudden infant death in this population as compared with the general population, and much of this increased risk is attributed to sleep disordered breathing. Although this risk decreases between 1 and 4 years of age, it is still increased in comparison with normal controls [71]. Craniofacial abnormalities in achondroplasia put these patients at elevated risk of OSA. FMS and ventriculomegaly are associated with increased risk of CSA, but a causal relationship has not been established. There was no direct correlation found between polysomnography findings and foramen magnum diameter. The incidence of CSA was not lower in children who had previously undergone spinal decompression; therefore, there may be other factors driving CSA in these patients. The prevalence of OSA in achondroplasia varies from 10% to 93% [69,74].

#### 2.6.4. Management

In order to prevent infant death in achondroplasia, polysomnography should be obtained within the first year of life. It can be difficult to distinguish physiologic apneas from pathologic central apneas during the infant period; therefore, evaluation should be obtained in a sleep lab experienced with children and with the ability to measure oxygen saturation, carbon dioxide levels, and EEG [74]. Children with OSA and achondroplasia should be evaluated for adenotonsillar hypertrophy, and adenoidectomy vs. adenotonsillectomy should be considered. In age appropriate children, removal of both adenoids and tonsils was shown to be superior to removal of adenoids alone. Residual OSA is common after surgical intervention. A follow up sleep study is recommended 2–4 months after the procedure to assess the need for continued CPAP use, particularly if the patient demonstrated severe OSA on pre-operative sleep study. Central apnea can also be seen in this group. Although the relationship between FMS and sleep disordered breathing is debated, patients with achondroplasia and central apnea should be evaluated for posterior fossa decompression or surgical therapy for ventriculomegaly [75].

All infants with achondroplasia have narrowed foramina magna compared with unaffected infants [76]. Relatively few infants with achondroplasia, however, require surgical decompression. An expert panel of skeletal dysplasia in 2015 had consensus agreement on two scenarios in which surgical decompression would be indicated for MRI-defined FMS: (1) FMS with cord signal change and (2) FMS with indentation of the spinal cord combined with abnormal neurological findings including observed apneic events and/or a disturbed sleep pattern with central events [70].

#### 2.6.5. Research and Future Directions

The increased risk of early childhood sudden death is a severe complication of achondroplasia, but the mechanism of this association is incompletely understood. Further research needs to be done to delineate the contribution of sleep disordered breathing versus other etiologies so that targeted and timely therapies can be employed. Additionally, further investigation is needed into how sleep disorders are modifying neural connectivity and leading to neuropsychological, cognitive, and behavioral dysfunction that can be difficult to manage.

### 2.7. Mucopolysaccharidoses

#### 2.7.1. Overview of Genetics

Mucopolysaccharidoses (MPS) are a collection of rare, inherited, lysosomal storage diseases known as Types I–IX. Depending on the specific enzyme deficiency, each type is known by a different name: MPS I as Hurler or Scheie syndrome (OMIM #607014), MPS II as Hunter syndrome (OMIM #309900), MPS III as Sanfillipo syndrome with four subtypes (OMIM #252900, OMIM #252920, OMIM #252930, OMIM #252940), MPS IV as Morquio syndrome (OMIM #253000), MPS VI as Maroteaux-Lamy syndrome (OMIM #253200), MPS VII as Sly syndrome (OMIM #253220), MPS IX as hyaluronidase deficiency (OMIM #601492) [77,78,79]. These disorders demonstrate autosomal recessive inheritance with the exception of MPS II, which demonstrates X-linked inheritance [79]. Collectively, they occur once in every 20,000 live births [77,80].

#### 2.7.2. Diagnosis and Molecular Genetics

Each type of MPS is caused by dysfunction of a specific enzyme involved in the degradation and metabolism of glycosaminoglycans (GAGs). Normally, GAGs are linear, unbranched polysaccharides on the cell surface or in the extracellular matrix and function in hydration homeostasis, regulation of growth and remodeling, and inflammation [81]. This causes accumulation of metabolic by-products that lead to cell injury, organ dysfunction, and reduced life expectancy [81]. Enzyme deficiencies are as follows: MPS I—alpha-l-iduronidase (OMIM #252800), MPS II—iduronate sulfatase (OMIM #300823), MPS III—four types with deficiency in heparan *N*-sulfatase proteins (OMIM #605270, #609701, #610453 and #607664), MPS IV—*N*-acetylgalactosamine-6-sulfate sulfatase (OMIM #611458), MPS VI—*N*-acetylgalactosamine-4-sulfatase deficiency (OMIM #611542), MPS VII—β-glucuronidase (OMIM #611499), MPS IX—hyaluronidase-1 (OMIM #607071) [7,77,78,82,83,84]. Due to the heterogeneity of presentation, diagnosis of MPS disorders are made based on clinical features, urinary and tissue GAG levels, enzyme assays, and genetic analysis [81].

#### 2.7.3. Clinical Characteristics

Specific system involvement is dependent on specific enzyme deficiency but may involve nervous system, musculoskeletal system, heart, liver, lung and airway, and otorhinolaryngologic manifestations. Typical clinical features are characterized by coarse facial features (with protruding or depressed frontal bone, depressed base of the nose, and/or hypoplastic mandible), macroglossia, restricted temporomandibular joint (TMJ) mobility, cognitive impairment, speech and language difficulties, hydrocephalus, spinal cord compression, seizures, frequent sinusitis and respiratory infections, adenotonsillar hypertrophy, airway GAG deposits, disorders of sleep, organomegaly, short stature, scoliosis, gait disturbance, and premature death in the second or third decade of life [7,77,80,81,82,85,86].

##### Developmental Aspects

Most patients with MPS have severe, progressive cognitive impairment; however, some patients with the mild form of MPS I (Scheie syndrome), MPS IV (Morquio syndrome), and MPS VI (Maroteaux-Lamy syndrome) may have milder cognitive impairment [81,85]. Many patients also have impairment of hearing and vision, speech and language development, and behavioral problems. Behavioral problems may manifest as temper tantrums, hyperactivity, and aggressiveness, which may be exacerbated by poor sleep quality [7,8].

#### 2.7.4. Sleep Disorders

##### Sleep Complaints

Due to diverse clinical features of MPS, sleep related complaints in MPS are varied and heterogeneous. Sleep disturbances that are reported in MPS include bedtime resistance, difficulty falling asleep, frequent awakenings often with disruptive behavior, parasomnias, excessive daytime sleepiness, snoring, and sleep disordered breathing [7,8,77,85]. Sleep disturbances have been associated with increased behavioral problems, depression, and reduced quality of life [7].

##### Sleep Architecture

Sleep architecture is variable in patients with MPS, and most of the studies investigating polysomnography in MPS have a small sample size and limit the study population to one or a few MPS types; however, collectively, sleep architecture disturbances that have been reported in MPS include prolonged sleep onset latency, which increased with age, decreased nighttime sleep, inefficient sleep, increased time in stage 1 sleep, less REM and slow wave sleep, and increased daytime sleep [7,78,87]. Sleep architecture disturbances may progress and worsen with age [7].

##### Circadian Rhythm 

It is unclear what contribution circadian rhythm disturbances may make to the disruption of sleep architecture, but this has been most extensively studied in MPS III [7,8,80]. Patients with MPS III may have elevation in daytime melatonin release, depression of nighttime melatonin, and reduced circadian variability which has also been demonstrated in mouse models of MPS III [7,8,80]. Although the mean periodicity of the circadian rhythm did not significantly differ between patients with MPS and typically developing controls, there was a greater variance and fragmentation of the circadian rhythm as measured by actigraphy in patients with MPS. Some MPS patients exhibited phase advancement, and some patients exhibited phase delay with phase delay being more common [80]. Descriptive activity analysis revealed increased activity from 12 a.m. to 6 a.m. and decreased activity level from 6 a.m. to 12 p.m. [80]. Disruption in the circadian rhythm can have significant impact on cognitive functioning, behavioral disturbances, and quality of life for patients and their caregivers [7]. In a pre-clinical murine study by Canal et al., they suggest that abnormal circadian responses in MPS III are a result of an alteration in the photo-retinal signaling to light rather than altered central nervous system (CNS) responses in the suprachiasmatic nucleus of the brain [8].

##### Sleep Related Breathing

One of the most commonly reported sleep disturbances in patients with MPS is sleep disordered breathing. Most patients with MPS reportedly snore and many suffer from OSA [77,79,86,87,88,89,90]. Patients with MPS I, II, or more severe forms of MPS are more likely to suffer from moderate to severe OSA, while patients with MPS I may suffer from lower oxygen saturation nadir than other forms of MPS [78,79,87,88]. Typical anatomic features of MPS that contribute to increased upper and lower airway obstruction include turbinate hypertrophy, adenotonsillar hypertrophy, decreased motion of the TMJ, macroglossia, increased deposition of GAG products throughout the upper and lower airway, laryngomalacia, subglottic stenosis, tracheomalacia, and restrictive lung disease [81,86]. It has been suggested that there is poor correlation between symptoms of sleep apnea and the presence of OSA on polysomnography. Therefore, patients with MPS should undergo PSG early in life [86]. There is variable evidence as to whether OSA severity is increased in young children or post-pubertal children and adults [87,90]. Restrictive lung disease in some patients with MPS may also put them at increased risk for hypopneas and sleep related hypoventilation with hypoxemia and hypercapnia, and this appears to worsen with age [78,81].

##### Movements and Nocturnal Events

Although not consistently reported in the literature of polysomnographic findings of patients with MPS, Lin et al. did record a higher periodic limb movement score in patients with MPS as compared with controls [87].

#### 2.7.5. Management

The focus of treatment of sleep disorders in MPS is centered on behavioral modification, regulation of the circadian rhythm, and treatment of sleep disordered breathing. As sleep apnea can worsen EDS and circadian rhythm disturbances, patients should undergo evaluation for sleep apnea using polysomnography if able, upon diagnosis. In the event that behavioral or cognitive impairment prohibits an evaluation using polysomnography, overnight oximetry may be an acceptable substitute. Patients with mild to moderate sleep apnea should undergo evaluation for airway obstruction and referral to ENT for possible adenotonsillectomy (T & A) [81]. In patients being considered for T & A, consideration of increased post-operative complications should be made with increased risk for post-operative hemorrhage, airway edema, and failure to extubate. Odontoid hypoplasia increases the risk of cervical instability, limited TMJ mobility, and upper airway obstruction and could lead to difficult intubation or preclude a successful procedure. Preparations should be made for the need for fiberoptic intubation and tracheotomy as needed [81,86]. Adenotonsillectomy is often successful in reducing the severity of OSA, but often insufficient to eliminate OSA completely. A post-operative sleep study should be obtained to assess the need for continued treatment of OSA with positive airway pressure (CPAP or BiPAP), or possibly tracheotomy placement. Although there is evidence of recurrence of adenoid hypertrophy following T & A in patients with MPS, the size of recurrent post-op adenoids is likely less than the pre-op adenoid size [86]. Newer treatment options have been used to prevent or minimize progressive disease including airway complications of MPS. Such treatments include enzyme replacement therapy or hematopoietic stem cell transplantation, yet the effects of these treatments on sleep related issues is unknown.

#### 2.7.6. Research and Future Directions

Given recent novel treatment approaches for MPS, it is unclear how these therapies such as intravenous or intrathecal enzyme replacement or bone marrow transplant alter the natural history of sleep disorders related to MPS progression. Longer term follow up of these patients will be needed to determine evolution of course and treatment for sleep disordered breathing.

### 2.8. Duchenne Muscular Dystrophy

#### 2.8.1. Overview of Genetics

Duchenne muscular dystrophy (DMD) (OMIM #310200) is an X-linked, recessive disorder involving a mutation in the dystrophin gene *DMD* (OMIM #300377) on Xp21. Due to its large size, the dystrophin gene is prone to spontaneous mutations (though most cases are inherited) and affects 1 per 3500 live male births [91,92].

#### 2.8.2. Diagnosis and Molecular Genetics

Dystrophin is a cytoskeletal protein that provides structure and function of cardiac and skeletal muscle by linking the internal actin cytoskeleton (F-actin) to the sarcolemma. Mutations in this gene cause muscle destruction, dysfunction, and muscular weakness [93,94]. Most patients with DMD have deletion or duplication of the dystrophin gene. Diagnosis is typically made with clinical presentation of progressive weakness in earlier childhood, using elevation of a creatine kinase level as screening and confirmation of DNA tests (deletion/duplication analysis with reflex to sequencing if negative) that has a 90–95% detection rate for DMD.

#### 2.8.3. Clinical Characteristics

Typical clinical characteristics of DMD include progressive weakness of skeletal muscles (including the diaphragm) and cardiac muscles with onset in early childhood around three to six years of age. Early signs may involve gait difficulty, frequent falls, muscle weakness of the proximal muscles greater than distal muscles, pseudohypertrophy of the calf presenting at about age 6, the need for a wheel chair for mobility around the age of 10–13 years, and chronic respiratory insufficiency and cardiomyopathy leading to premature death in the second to third decades of life [92,93]. Once non-ambulatory, most patients develop scoliosis. Due to muscular weakness, respiratory hypoventilation and sleep disordered breathing are significant problems for children with DMD. Unfortunately, one quarter of unexpected deaths in DMD occur unexpectedly at night [91].

##### Developmental Aspects

Children are typically normal at birth and may only exhibit slight delay until increased motor weakness becomes evident during preschool ages. Children with DMD are at risk for neurodevelopmental impairment. One study of 130 males with DMD showed that intelligence was below the population mean, with intellectual disability observed in 34 males (26%). Over one third of males had abnormal scores on more than two measures of emotional, behavioral, or neurodevelopmental problems. Mutations in the 3′ end of the large dystrophin gene are considered to affect more isoforms of dystrophin which is expressed in patients with DMD. Higher rates of intellectual disability and social cognitive dysfunction were found in males with mutations at the 3′ end of the *DMD* gene [95].

#### 2.8.4. Sleep Disorders

##### Sleep Complaints 

Children with DMD are at high risk for sleep disordered breathing including OSA or sleep-related hypoventilation. Clinical symptoms may include snoring, morning headaches, hypersomnolence, lethargy, fatigue, nonrestorative sleep, sleep-related hyperhidrosis, and attention difficulties. Many of these symptoms may overlap with symptoms of neuromuscular weakness. Several reports have suggested poor correlation between patient-reported symptoms and the presence of sleep disordered breathing in this population [93,96,97,98].

##### Sleep Architecture

One study investigated sleep architecture in patients with DMD compared with control subjects without neuromuscular disorders undergoing polysomnography for other sleep related complaints. They identified changes in sleep quality such as decreased sleep efficiency, increased REM latency, decreased percentage of REM sleep, and increased wake after sleep onset [93,96].

##### Sleep Related Breathing

**Figure 2:**
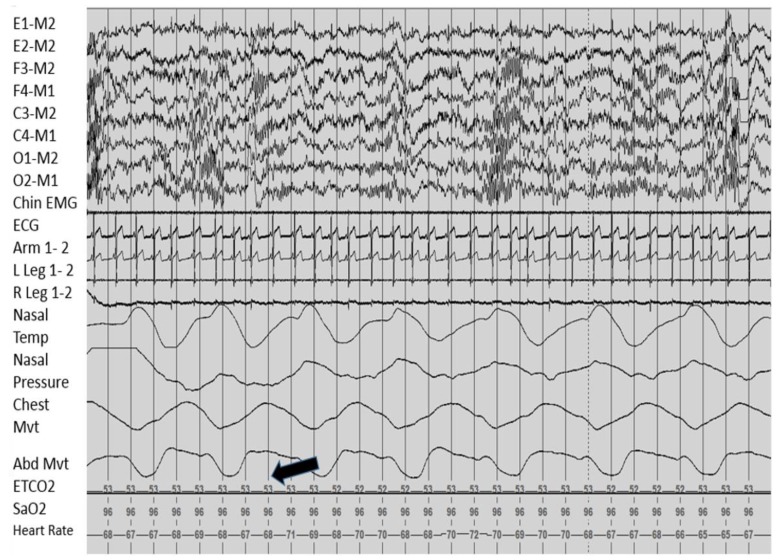
Hypercapnia in muscular dystrophy. This figure, from a polysomnogram of a child with Duchenne muscular dystrophy, shows persistent elevated CO_2_ levels during sleep noted by the arrow.

Polysomnography is the gold standard for evaluation of sleep disordered breathing in children with DMD. Overnight oximetry may identify sleep related hypoxemia, but given concern for hypoventilation, a sizeable proportion of hypoventilation may be missed by oxygen monitoring alone. Therefore, it is recommended that sleep assessment in DMD patients includes end tidal CO_2_ monitoring or transcutaneous CO_2_ in children. One study in patients with median age of 10 suggested that the use of pediatric criteria for diagnosis of sleep disordered breathing increased the likelihood of receiving a diagnosis of sleep disordered breathing [96]. The sleep study should be done in a location with expertise in performing and scoring pediatric sleep studies and with the ability to monitor CO_2_ levels [99,100]. Figure 2 as shown, demonstrates the significant hypercapnia that may be observed during overnight polysomnography of a patient with DMD.

OSA is the predominant phenotype of sleep disordered breathing at younger ages and sleep-related hypoventilation is present in most patients with DMD at older ages, but there is significant overlap given risk factors for obesity and variability in progression of muscle weakness [96,97]. Obesity has been associated with worsened OSA. Additionally, obstructive apnea index in patients with DMD is worse in REM sleep when compared to non-REM sleep [97], which probably is related to diaphragm weakness in this population. In one study classifying patients as OSA, CSA, or sleep related hypoventilation, patients with an increased forced vital capacity (FVC) had increased odds of OSA while patients with a reduced FVC had increased odds of hypoventilation [97]. Steroid therapy in young boys with DMD may contribute to increased obesity and OSA prevalence prior to the onset of sleep related hypoventilation [97].

CSA has been shown to occur in up to 33% of patients with DMD as well [97]. It is unclear from the evidence whether these central events are truly central events or are classified as central events on polysomnography due to poor signaling in the setting of decreased respiratory muscle strength [97].

As patients with DMD get older and respiratory muscle function decreases, they are at increased risk of nocturnal, and eventually diurnal, hypoxemia and hypoventilation. Older patients with a FVC less than 1820 mL are at increased risk for nocturnal hypercapnia, and those with a FVC of 680 mL or less have an increased risk for daytime hypercapnia as well [101]. We know that neuromuscular and diaphragm weakness in individuals with DMD can contribute to nocturnal hypoventilation, hypoxemia, and sleep disordered breathing. In a mouse model of DMD, it was found that DMD mice exposed to episodic hypoxemia as a model of sleep disordered breathing had further reduced respiratory muscle strength and ventilatory dysfunction. The mechanism of this association is not known, but suggested mechanisms include increased mechanical fragility to increased respiratory muscle recruitment during periods of hypoxemia, impaired cellular repair mechanisms, and increased oxidative stress [102,103].

##### Movements and Nocturnal Events

Leg movement index has been shown to be increased in DMD children as compared to healthy controls and was further increased in wheelchair-bound patients [98,104]. This is an expected finding given that patient with neuromuscular weakness must ask for repositioning if unable to accomplish the movements themselves, but it does significantly contribute to worsened sleep quality of patients and their caregivers.

#### 2.8.5. Management

Although many DMD patients with sleep disordered breathing report clinical symptoms and many have restrictive lung disease evident on spirometry and plethysmography, these are not adequate screening tools for sleep disordered breathing in older children with DMD [96,100]. An FVC less than 40% has been shown to be sensitive, but not specific, for sleep related hypoventilation as defined by elevated CO_2_. An FVC less than 40% also did not correlate with AHI [105]. Non-ambulatory patients are at high risk for sleep disordered breathing. Therefore, nocturnal polysomnography should be obtained for any patient with symptoms of sleep disordered breathing, significant drop in FVC, FVC less than 40–60% of predicted values for normative values, or non-ambulatory patients. Patients with DMD likely benefit from early identification and treatment of sleep disordered breathing [91,97,100,103,105].

Systemic corticosteroids are a mainstay of treatment for DMD early in childhood, and the use of steroids puts children at increased risk of obesity and OSA. Sleep related breathing should be monitored closely during therapy with systemic steroids [92]. It has been suggested that melatonin plays a role in vitamin D deficiency, which in turn, can impact somnolence and sleep disordered breathing. Therefore, monitoring vitamin D status particularly during systemic steroid treatment may be important for patients with DMD [92].

Younger patients with OSA may benefit from surgeries to promote airway patency such as adenotonsillectomy. This may defer the need for positive airway pressure, but due to the risk for residual hypoventilation post-operative sleep study should be obtained [93].

Noninvasive positive-pressure ventilation (NIPPV) is one of the most important treatments in recent decades for DMD patients, and its use has increased the median survival of patients with DMD by 10 years and improved quality of life. Positive airway pressure (PAP) treatment in patients with DMD has been shown to improve perceived symptoms [65,91,106]. Although CPAP may be an effective treatment for DMD patients with OSA or early in the disease course, eventually, all patients with DMD will develop sleep related hypoventilation and require bilevel therapy so the use of BiPAP at the onset of sleep disordered breathing may avoid the need to switch devices later in disease course [91]. Caution should also be used in the consideration of supplemental oxygen for sleep related hypoxemia in patients with DMD. It has been shown that although the use of supplemental oxygen in patients with advanced DMD may improve oxygenation, it may significantly prolong hypopnea and hypoventilation duration and severity [107].

#### 2.8.6. Research and Future Directions

Despite significant technical advancements in PAP delivery modalities, life expectancy for young men with DMD is very limited and the transition to more prolonged use of mechanical ventilation can be a very challenging decision based on the patient’s values and goals; however, it is the mainstay for treatment of progressive respiratory failure in these patients. Additionally, a novel therapeutic agent, eteplirsen (Sarepta Therapeutics, Cambridge, MA, USA) was recently approved by the Food and Drugs Administration (FDA), which targets splicing mutations in 13–14% of patients with DMD with mutations that involve exon 51 skipping. Although the clinical benefits of this therapy are unclear, it has slightly increased dystrophin levels in some patients and has a low side effect profile. The FDA has approved its use in patients with appropriate dystrophin mutations. There is questionable benefit in a clinical six-minute walk test over four years, but there is hope that it will delay progression of disease in some patients if started at young ages. Due to the lack of evidence, it is unclear how this will effect sleep disordered breathing in these patients, but hopefully this advancement will lead to novel therapies in treating DMD [108]. Other therapies targeting restoration of the dystrophin protein such as gene therapy are under investigation.

## 3. Conclusions

Sleep disorders are prevalent in children with neurogenetic disorders. In addition to the risk of sleep disordered breathing, patients with neurogenetic disorders are often at risk for other sleep disorders, such as disrupted intrinsic sleep structure in patients with AS, disrupted circadian rhythm in patients with SMS, and hypoventilation related to diaphragm and neuromuscular weakness in patients with muscular dystrophies. Disruption of sleep structure, onset, maintenance, and quality in any form can significantly impact patient and caregiver quality of life. If identified, sleep disruptors are often treatable. Untreated sleep disruptions may lead to worsening of neurocognitive or behavioral manifestations that make the care of patients with neurogenetic disorders very challenging. Identifying, diagnosing, and treating these sleep disorders may significantly improve patient and caregiver quality of life. It may also prolong survival or prevent development of sequelae or comorbidities of untreated sleep disorders such as in OSA or chronic hypoventilation. Development of comorbid conditions can make chronic disease management very challenging for patients, providers, and families. Optimal quality sleep is important in neurocognitive and psychological functioning and development. It is important to screen patients with neurogenetic disorders for sleep disturbances and poor sleep quality during routine health maintenance examinations. Treatment of sleep related disorders in patients with these complex and often multi-system diseases are often best managed in a multi-disciplinary setting with primary care, sleep physicians, pulmonary, neurology, ENT, respiratory therapy, and nursing as needed.

## Figures and Tables

**Table 1:** Neurogenetic disorders included in this review.

Suspected Localization of Pathogenesis of Sleep Disorder	Neurogenetic Disorders
**Multi-system** Brainstem/orofacial structure/airway/chest	Down syndrome (DS)
**Central nervous system** Brain Brain (hypothalamus) Brain Brainstem/autonomic system	Angelman syndrome (AS) Prader–Willi syndrome (PWS) Smith–Magenis syndrome (SMS) Congenital central hypoventilation syndrome (CCHS)
**Skeletal/spinal cord** Medullary-cervical cord Upper airway/cervical cord	Achondroplasia/hypochondroplasia Mucopolysaccharidoses (MPS)
**Peripheral nervous system** Muscles	Duchenne muscular dystrophy (DMD)
